# Local C-Reactive Protein Expression in Obliterative Lesions and the Bronchial Wall in Posttransplant Obliterative Bronchiolitis

**DOI:** 10.1155/2009/510254

**Published:** 2009-05-26

**Authors:** Outi E. Päiväniemi, Paula K. Maasilta, Tiina L. S. Vainikka, Hanni S. Alho, Pekka J. Karhunen, Ulla-Stina Salminen

**Affiliations:** ^1^Department of Cardiothoracic Surgery, Helsinki University Hospital, P.O. Box 340, 00029 Helsinki, Finland; ^2^Department of Orthopedics and Traumatology, Tampere University Hospital, P.O. Box 2000, 33521 Tampere, Finland; ^3^School of Medicine, Tampere University, 33104 Tampere, Finland; ^4^Research Unit, Centre of Laboratory, Tampere University Hospital, P.O. BOX 2000, 33521 Tampere, Finland

## Abstract

The local immunoreactivity of C-reactive protein (CRP) was studied in a heterotopic porcine model of posttranplant obliterative bronchiolitis (OB). Bronchial allografts and control autografts were examined serially 2–28 days after subcutaneous transplantation. The autografts stayed patent. In the allografts, proliferation of inflammatory cells (*P* < .0001) and fibroblasts (*P* = .02) resulted in occlusion of the bronchial lumens (*P* < .01). Influx of CD4+ (*P* < .001) and CD8+ (*P* < .0001) cells demonstrated allograft immune response. CRP positivity simultaneously increased in the bronchial walls (*P* < .01), in macrophages, myofibroblasts, and endothelial cells. Local CRP was predictive of features characteristic of OB (*R* = 0.456–0.879, *P* < .05−*P* < .0001). Early obliterative lesions also showed CRP positivity, but not mature, collagen-rich obliterative plugs (*P* < .05). During OB development, CRP is localized in inflammatory cells, myofibroblasts and endothelial cells probably as a part of the local inflammatory response.

## 1. Introduction

Posttransplant obliterative bronchiolitis (OB) is the major cause of lung allograft dysfunction and late mortality among lung allograft recipients [1]. The registry data indicates that, 5 years after transplantation, 45% of recipients had developed OB [1]. OB is a manifestation of chronic allograft rejection with histopathological features that include subepithelial inflammation, epithelial cell injury, and excessive fibroproliferation, resulting in permanent obliteration of small airways [2]. Long-term success in lung transplantation will only be possible when there is better understanding of the molecular and cellular mechanisms of OB and better strategies for its treatment. 

C-reactive protein (CRP) is an acute-phase protein that is predominantly synthesized by hepatocytes in response to inflammation, infection, and tissue damage [3, 4]. Extrahepatic synthesis of CRP was first described in human peripheral blood lymphocytes [5]. Expression in human neurons [6], carotid arterial endothelial cells [7], normal vascular tissue [8], coronary artery smooth muscle cells [9, 10], atherosclerotic plaques [11, 12], and adipocytes [13] has also been reported. In the human respiratory tract, epithelial cells in nasal polyps express CRP [14], as do alveolar macrophages in lung tissue [15].

CRP is used as a clinical marker of acute systemic inflammation. Pulmonary diseases with inflammatory features raise the serum CRP level [16, 17], and there is also evidence of local pulmonary CRP synthesis [18]. Since OB has inflammatory features, we hypothesized that local CRP production could occur in posttransplant OB development. To investigate this hypothesis, we conducted a detailed CRP expression analysis in our porcine model of posttransplant OB [19]. 

## 2. Materials and Methods

### 2.1. Experimental Model

Bronchial segments were transplanted subcutaneously in 11 random-bred unrelated domestic pigs, each weighing 20 kg. The animals received humane care in compliance with the “Principles of Laboratory Animal Care” (NIH publication Vol 25, No. 28 revised 1996) and with all specific national legislation. The protocol was approved by the institution's Committee for Animal Research and by the South Finland Provincial Administration. Special attention was given to anesthesia and pain relief during surgical procedures.

Animals were anesthetized for surgical procedures as described previously [20] and euthanized with high-dose intravenous sodium pentobarbital at the end of the follow-up. The donor left caudal pulmonary lobe was removed for preparation of implants. A series of bronchial segments, 1.5 cm in length, were subcutaneously transplanted into the ventral side of each recipient. In addition to 6 animals with allograft implants, 5 animals received autograft controls. A total of 190 samples were harvested through small incisions on follow-up days 2, 4, 7, 9, 11, 14, 17, 21 and 28. A portion of each sample (two-thirds) was formalin-fixed and embedded in paraffin, and the rest (one-third) was snap-frozen in liquid nitrogen and stored at −7°C until use. 

### 2.2. Histology and Immunocytochemistry

The formalin-fixed sections (4 *μ*m) were mounted on Vectabond-coated slides (Vector Labs. Ltd., Peterborough, UK) and processed for histology (hematoxylin and eosin) and immunocytochemistry. Monoclonal mouse antipig antibodies (Sigma Chemical, St. Louis, MO, USA) against vimentin (1 : 1000) *α*-smooth muscle cell actin (*α*-SMA) (1 : 16 000) were used as described previously [21], along with standard avidin-biotin-peroxidase techniques [22]. 

The Frozen sections, 4 *μ*m thick, were cut and air-dried onto silane-coated slides, fixed in acetone at −20°C for 10 minutes and stored at −20°C until use. The 3-layer indirect immunoperoxidase method and monoclonal mouse antibodies detecting swine CD4 and CD8 (VMRD Inc., Pullman, Wash, USA) were used in addition to mouse monoclonal antibodies against human monocytes/macrophages (Oxford Biomarketing, Oxford, UK), also reactive with porcine macrophages, and against human CRP (Abcam Ltd., Cambridge, UK) (1 : 600), also reactive with porcine CRP, as described previously [23]. As a control, nonspecific mouse IgG (Dako A/S, Glostrup, Denmark) was used to stain all sections by an identical protocol.

### 2.3. Microscopic Assessment

Assessments were performed on 3 separate bronchi from each sample when available. Epithelial loss, luminal obliteration, bronchial wall fibrosis (a pathological increase in connective tissue composed of fibroblasts and extracellular matrix), and inflammation (the number of infiltrating inflammatory cells in the tissue) were graded on a semiquantitative scale. The scale, using scores ranging from 0 to 3, was as follows: 0 = no alteration, 1 = mild alteration, 2 = moderate alteration (pathological alterations equal to normal tissue), and 3 = severe alteration (pathological changes predominant).

CD4+ cells, CD8+ cells, and macrophages were counted in 3 high-power visual fields for each bronchus that was assessed. The percentages of CRP-positive cells in the epithelium and the obliterative plug were counted separately, and the staining intensity was graded on a scale from 0 to 3, where 0 = negative; 1 = positive cells showing weak staining; 2 = more intense and/or irregular staining; 3 = intense, uniform staining. The CRP index was calculated by multiplying the percentage of CRP-positive cells by the CRP staining intensity score, a method we previously used to assess cyclo-oxygenase expression [20]. CRP-positive cells in the bronchial wall were assessed on a scale from 0–3 based on the intensity of immunocytochemical staining and the number of positive cells. 

### 2.4. Statistics

All data are expressed as the mean ± standard error (SEM). The Mann-Whitney U-test, Z adjusted for ties (Statistica v.5.1, StatSoft Inc., Tulsa, OKla, USA), was used for statistical analysis, with *P* ≤ .05 considered statistically significant. Spearman's rank correlation served for correlation analysis.

## 3. Results

### 3.1. Histology

On posttransplant day 2, ischemic damage to the airway epithelium was moderate both in the autograft controls (grade 1.8 ± 0.3) and in the bronchial allografts (grade 1.7 ± 0.2). Rapid recovery to normal respiratory epithelium (94%; *P* < .0001) occurred in the autografts within 7 days. In allografts, temporary and partial recovery on day 4 (grade 0.7 ± 0.2) turned to rapid loss of the epithelial cells with grade 2.5 ± 0.1 on day 7, when a significant difference (*P* < .001) was apparent compared to autografts; at this time, most of the preserved epithelium comprised the pure basal cell layer (88%; *P* < .0001) in allografts.

**Figure 1:**
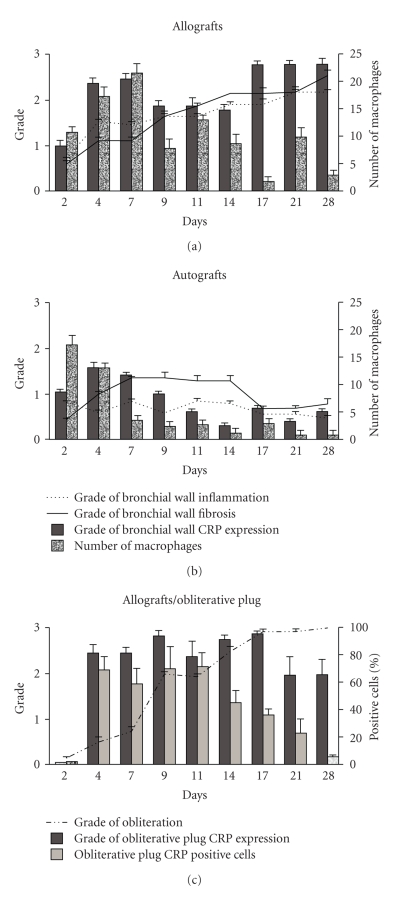
Bronchial wall alterations in bronchial allografts and autografts and alterations in the obliterative plug. Variables determining the histological grade of inflammation and fibrosis of the bronchial wall are given for bronchial allografts and autograft controls together with the intensity of immunocytochemistry against CRP, all on a scale from 0–3. In addition, the number of macrophages (MP) in the bronchial wall is given for allografts and autografts. Cells are counted per high power visual field. The histological grade of luminal obliteration (0–3) is given for allografts together with the percentage of cells positive for CRP in the obliterative plug and with the intensity score of the staining. Autografts remained patent with no observed obliterative plugs.

Inflammation in the bronchial wall remained low grade in the autografts (Figure 1). In allografts, the number of inflammatory cells gradually increased, and there was a significant difference compared to autografts from day 4 (*P* < .0001) on. The onset of fibroproliferation in the bronchial wall paralleled the increase in inflammation grade, and fibroproliferation was significantly different in allografts compared to autografts by day 11(*P* = .02) (Figure 1). Obliterative lesions gradually occluded the bronchial lumens in allografts, showing a significant difference from autografts from day 7 (*P* < .01) on (Figure 1). Severe remodeling of the bronchial allografts occurred, while autografts stayed patent with functioning mucus glands. 

### 3.2. Immunocytochemistry

Fibroblasts showed positive staining for vimentin and *α*-SMA both in the bronchial wall and in the obliterative lesions. Intense positivity for *α*-SMA was evident in newly formed obliterative plugs, but not in the mature, collagen-rich, scar-like lesions.

In the bronchial wall of the control autografts, a few CD4+ and CD8+ cells were observed throughout follow-up. An influx of CD4+ and CD8+ cells with CD8+ cell predominant indicated an immune response in the bronchial allografts that was significantly different from autografts from day 4 onward (Table 1). The number of macrophages was highest during the initial ischemic injury and at the onset of the immune response; macrophages were present in greater numbers from day 7 (*P* < .0001) onward in allografts, showing a statistically significant difference also on days 9 (*P* < .0001), 11, 14, 21, and 28 (Figure 1).

During the initial ischemic damage on follow-up day 2, 57% of the respiratory epithelial cells in the autografts and 48% in the allografts expressed CRP. The CRP staining intensities were 2.76 ± 0.2 and 2.0 ± 0.2, and the CRP indexes were 151 ± 22 and 103 ± 20, respectively. CRP expression rapidly decreased in parallel with epithelial damage in the allografts: 16% (*P* < .0001) of preserved cells had a CRP staining intensity of 0.9 ± 0.5 (*P* < .01) and a CRP index of 46 ± 31 (*P* < .001) on day 7. Moderate to intense epithelial expression of CRP was observed throughout follow-up in the recovered respiratory epithelium of autografts. 

**Figure 2:**
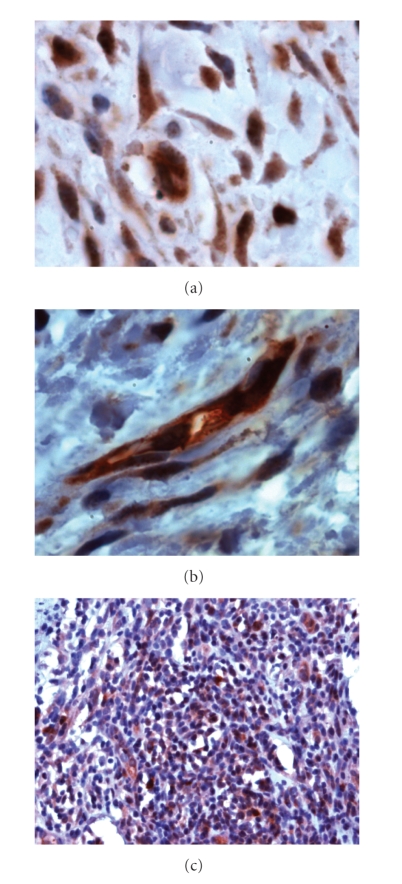
Bronchial wall alterations in bronchial allografts. Macrophages, fibroblasts (a) and endothelial cells (b) stained postive for CRP in the bronchial wall. CRP positivity was abundant in fresh obliterative plugs on the follow-up day 11 (c). Immunocytochemistry against CRP: hematoxylin counterstaining, original magnification 100x objective (a, b), and 20x objective (c).

CRP expression was observed in all implants in the bronchial wall during initial ischemic damage, and then decreased in the autografts. In contrast, expression rapidly increased in the bronchial allografts (Figure 1). A statistical difference between autografts and allografts was reached on follow-up day 9 (*P* < .01). Expression was not only first predominantly observed in macrophages (Figure 1), but also observed in myofibroblasts and endothelial cells (Figure 2). While the number of macrophages decreased towards the end of follow-up, the intensity of their staining increased as did endothelial CRP immunoreactivity. Positive myofibroblasts counted a minority and were observed at the beginning of follow-up. Correlation analysis showed that the grade of CRP staining intensity in the bronchial wall on follow-up days 14, 17, 21, and 28 appeared to be correlated with and predictive of bronchial wall inflammation, the number of CD4+ and CD8+ cells, bronchial wall fibroproliferation and luminal obliteration (Table 2). These are all features that are characteristic of OB. 

In the early obliterative lesions, the majority of cells including fibroblasts, inflammatory cells, and endothelial cells showed CRP-positivity with ≥2.0 intensity scores (Figure 1). After day 11, when 72% of the cells in the obliterative plug were CRP-positive, the percentage of cells expressing CRP rapidly decreased. The calculated CRP index was 175 ± 38 in the early obliterative lesions on day 4, but only 18 ± 4 in the mature, collagen-rich plugs on day 28. There was a statistically significant difference in the percentage of positive cells and in the CRP index on day 9 compared to day 17 as well on day 14 compared to day 28 (*P* < .05). The controls stained negative.

## 4. Discussion

This study shows that CRP is expressed in the bronchial wall and in obliterative lesions predominantly not only by macrophages but also by myofibroblasts and endothelial cells in posttransplant OB development. Local CRP expression was previously observed in respiratory epithelial cells [14, 24] and in alveolar macrophages [15, 25]. To our knowledge, this is the first report of CRP expression in pulmonary fibroblasts and endothelial cells in the lung.

In our porcine model, OB developed invariably in allografts, while autografts stayed patent, as expected [19, 26]. The histological changes were similar to those seen in human OB [2, 27] and in rodent models [28, 29]. Neovascularization is rapid in the implants [19]; thus, the initial ischemic injury totally recurred within one week in autografts. In addition, partial recovery prior to onset of the immune response was also observed in the allografts. The influx of CD4+ and CD8+ cells was indicative of the cell-mediated immune response [30]. Vimentin and *α*-SMA postitivity indicated myofibroblasts in the fibroproliferatory stage both in the bronchial wall and in the obliterative lesions [31] and macrophages stained positive with specific immunocytochemistry.

CRP expression has previously been reported in corticular tubules and glomerular cells in rejecting kidneys [32]. Measurement of urine CRP in renal transplant recipients is considered a useful tool in the diagnosis of early allograft rejection [33]. CRP also has properties to serve as a sensitive marker of lung injury [25]. However, our study does not support the use of CRP measurement in the lavation fluid as an early marker of OB, because local CRP is also expressed in human lungs in other inflammatory conditions in response to inflammatory stimuli [24, 34]. Similarly, in our study autografts constantly produced CRP in the respiratory epithelium. This probably occurred initially as a response to the ischemic operative injury and later on as a response to the constantly increasing pressure inside the bronchial implants due to intraluminally secreted mucus. We suggest that the epithelial expression of CRP in autografts is characteristic of this model. This was also previously observed in induced nitric oxide synthase and nitrotyrosine expression [35].

Paralleling the immune response in allografts, we observed CRP expression in the bronchial wall in macrophages and also in myofibroblasts and endothelial cells. Alveolar macrophages are known to express CRP [15]. CRP expression has previously been observed also in endothelial cells [7] and smooth muscle-like cells [9, 10]. In the present study, the intensity of staining increased during follow-up in macrophages and endothelial cells, but was observed only in early myofibroblasts as previously in smooth muscle-like cells [7] and in early fibrous plaques [36]. At the end of the follow-up macrophages stained intensely, as did the endothelium of the numerous capillaries. The grade of CRP intensity in the bronchial wall correlated with and was predictive of further inflammation, the number of CD4+ and CD8+ cells, and fibroproliferation, including obliteration of the bronchial lumen. All of these features are characteristic of OB [37]. In autografts, mural CRP production was turned off. However, CRP expression was observed in autografts during the first days of follow-up, when ischemic damage recurred, and subsequent inflammation was established. Endothelial CRP production has been reported in human carotid arteries [7] and in the aorta [38], but it has not previously been reported in lung tissue or in cases of lung allograft rejection. In this study, CRP expression coincided with the remodeling process and with bronchial wall inflammation as it has previously been reported to associate with the progression of atherosclerosis in coronary arteries [36]. 

Inflammatory cytokines interleukines (ILs) 1 and 6 are stimulatory, but tumor necoris factor (TNF) is an inhibitory cytokine for the liver synthesis of CRP [39]. Local CRP expression is also thought to be a response to inflammatory cytokines [9] which regulate the immune cascade in lung allograft rejection. In renal tubular epithelial cells, TNF-*α* does not stimulate local CRP expression [32], but in human pulmonary epithelial cells, CRP is synthesized in response to cytokines IL6, TNF-*α*, interferon *γ*, and IL1*β* [24]. Adipocytes are also shown to produce CRP under the influence of IL6 and TNF-*α* [13]. Casals et al. suggested that CRP could modulate lung inflammation by decreasing the production of TNF-*α* in alveolar macrophages [25]. We have previously studied TNF-*α* in our model of OB [40]. In the bronchial wall its expression paralleled the immunoreactivity of CRP. TNF-*α* expression seems to decline prior to that of CRP during obliterative airway disease development. This might be a consequence of stimulating influence of the TNF-*α* on CRP expression. 

In the early obliterative plug, CRP positivity was observed in the majority of cells. In the mature plug expression declined significantly. Thus, as in atherosclerotic lesions [38], local CRP in the obliterative plaque contributes with plaque activity. In maturing carotid artery plaques, CRP expression is evident in inflammatory cells as well as in the endothelial cells of newly formed microvessels [7]. Our study found CRP expression in early obliterative plugs, but not in the mature, scar-like lesions, in which the existence of *α*-SMA was also not expressed. The most intense CRP expression was observed during the accelerated inflammatory phase. Thus, it seems that CRP in obliterative plugs is a local inflammatory response, as in atherosclerotic lesions [38].

Inhibition of complement activation in OB development seems to have beneficial effects on graft survival [41]. There is evidence that CRP limits excessive complement activation [42] and reduces complement-mediated damage to host tissue. In addition, CRP and complement components act in concert to promote clearance of apoptotic cells, which prevents necrosis [43]. We have demonstrated that apoptotic cell death is an important mechanism in the process that leads to graft deterioration in OB after lung transplantation [44]. The exact role of CRP in our model of OB remains unresolved. It may be observed that local CRP has a promoting influence on the inflammatory component [45, 46] and also on allograft rejection. 

## 5. Conclusions

This study documents CRP immunoreactivity in posttransplant OB. There are multiple factors leading to chronic lung allograft rejection. CRP expression may be a local response to inflammatory cytokines and may promote the inflammatory component in OB development.

## Figures and Tables

**Table 1:** Immune cell influx in bronchial autografts and allografts. An early influx of CD4+ and CD8+ cells with CD8+ cell predominance indicates an immune response in the bronchial allografts.

Follow-up day	Autografts		Allografts	
	CD4+ cells	CD8+ cells	CD4+ cells	CD8+ cells
2	1.4 ± 0.2	2.9 ± 0.4	2.6 ± 0.5	^1^4.4 ± 0.5
4	5.5 ± 3.2	8.6 ± 1.5	^2^30.7 ± 4.4	^3^37.7 ± 3.7
7	9.2 ± 1.0	12.1 ± 1.7	^3^30.4 ± 3.2	^3^61.3 ± 4.7
9	4.0 ± 0.6	5.6 ± 0.6	^3^60.9 ± 3.9	^3^68.9 ± 5.0
11	8.1 ± 0.9	11.2 ± 1.4	^3^46.2 ± 3.6	^3^84.4 ± 5.3
14	8.5 ± 1.1	11.0 ± 1.5	^3^57.5 ± 5.4	^3^72.3 ± 6.2
17	3.1 ± 0.7	5.8 ± 0.9	^3^43.9 ± 4.5	^3^55.4 ± 5.2
21	6.3 ± 0.9	8.0 ± 0.9	^3^63.7 ± 5.2	^3^76.4 ± 5.6
28	6.7 ± 0.9	8.4 ± 0.6	^3^57.1 ± 8.4	^3^63.9 ± 5.6

^1^ *P* < .05, ^2^ *P* < .001, ^3^ *P* < .0001.

**Table 2:** Correlations (R) to the grade of bronchial wall CRP. R is given for assessment points with statistical significance (*P* < .05). The grade of CRP expression in the bronchial wall turned out to correlate to the inflammatory alterations and bronchial remodeling processes. Correlations are given from the follow-up day 14 onward. On the follow-up day 14 onward, grade of CRP expression was significantly (*P* < .0001) more intense in allografts than in autografts.

Mural inflammation	CRP/day 14	CRP/day 17	CRP/day 21	CRP/day 28
day 14	0.658			
day 17	0.823	0.900		
day 21	0.834	0.522	0.720	
day 28	0.879	0.590	0.693	0.716
N of CD4+ cells	CRP/day 14	CRP/day 17	CRP/day 21	CRP/day 28
day 14	0.764			
day 17	0.663	0.815		
day 21	0.480	0.532	0.669	
day 28	0.456	0.516	0.595	0.551
N of CD8+ cells	CRP/day 14	CRP/day 17	CRP/day 21	CRP/day 28
day 14	0.487			
day 17	0.741	0.874		
day 21	0.568	0.600	0.672	
day 28	0.528	0.475	0.689	0.591
Mural fibrosis	CRP/day 14	CRP/day 17	CRP/day 21	CRP/day 28
day 14	0.739			
day 17		0.864		
day 21	0.525	0.737	0.804	
day 28	0.642	0.926	0.841	0.747
Luminal obliteration	CRP/day 14	CRP/day 17	CRP/day 21	CRP/day 28
day 14	0.845			
day 17	0.795	0.797		
day 21	0.813	0.810	0.875	
day 28	0.811	0.848	0.868	0.870

## Abbreviations

OB: Obliterative bronchiolitis
CRP: C-reactive protein
*α*-SMA: *α*-smooth muscle cell actin
SEM: Standard error
IL: Interleukine
TNF-*α*: Tumor necrosis factor-*α*
